# Investigation of Reference Genes for qRT-PCR and ARF Gene Family in *Michelia compressa* (Magnoliaceae) Under Cold Stress

**DOI:** 10.3390/plants15091317

**Published:** 2026-04-25

**Authors:** Luomin Cui, Tong Wu, Zhiquan Wang, Xiaowei Sun, Jinhong Li, Zhiguo Lu, Chaoguang Yu, Fangyuan Yu, Yunlong Yin

**Affiliations:** 1 Co-Innovation Center for Sustainable Forestry in Southern China, College of Forestry and Grassland, College of Soil and Water Conservation, Nanjing Forestry University, Nanjing 210037, China; cui2022@njfu.edu.cn (L.C.); wttt@njfu.edu.cn (T.W.); 19989106159@163.com (J.L.); 2 Jiangsu Key Laboratory for Conservation and Utilization of Plant Resources, Institute of Botany, Jiangsu Province and Chinese Academy of Sciences (Nanjing Botanical Garden Mem. Sun Yat-Sen), Nanjing 210014, China; wangzhiquan@cnbg.net (Z.W.); sxw0836@foxmail.com (X.S.); lzgjs@cnbg.net (Z.L.); yuchaoguang168@cnbg.net (C.Y.)

**Keywords:** *Michelia compressa*, reference gene, cold stress, ARF transcription factor, functional analysis

## Abstract

*Michelia compressa*, a member of the Magnoliaceae family, is an evergreen tree of considerable significance in both landscape gardening and industrial production. However, during its introduction to northern subtropical regions in China, this species often suffers from frost damage, which limits its widespread application. The utilization of housekeeping genes is essential when performing gene family analyses under abiotic stress conditions. Additionally, auxin response factor (ARF) transcription factors (TF) play a crucial role in plant responses to abiotic stresses; however, their specific function in cold stress responses within *M. compressa* has not been systematically investigated. Ten housekeeping genes were selected from transcriptome data for evaluation using quantitative real-time PCR (qRT-PCR). The optimal housekeeping gene identified through screening was used for verification of gene family analysis. Additionally, key genes underwent functional validation. Analysis conducted with GeNorm, NormFinder, and BestKeeper identified *28S* as the optimal reference gene for *M. compressa* under cold stress. Furthermore, an analysis of the *ARF* gene family using full-length transcriptome data revealed a total of 48 *McoARF* genes, which clustered into three groups alongside the *Arabidopsis thaliana ARFs*. Among these, eight selected *McoARF* genes exhibited significantly elevated expression levels in leaves under cold stress and demonstrated tissue specificity. Functional validation revealed that transgenic plants overexpressing *McoARF13* displayed elevated levels of reactive oxygen species (ROS), hydrogen peroxide (H_2_O_2_), and malondialdehyde (MDA), as well as increased activities of peroxidase (POD) and superoxide dismutase (SOD) in leaves under cold stress. This study represents the inaugural screening of housekeeping genes in *M. compressa* under cold stress conditions, accompanied by an analysis of the *ARF* gene family. The functional validation of *McoARF13* was successfully conducted, offering valuable insights into the molecular mechanisms that underlie cold stress response in *M. compressa*.

## 1. Introduction

The genus *Michelia* (Magnoliaceae), comprising a diverse array of evergreen woody plants, plays a vital role in urban forestry by providing essential ecosystem services and aesthetic benefits. Characterized by their graceful architecture and fragrant blooms, *Michelia* species are extensively integrated into urban green spaces across northern subtropical regions in China to enhance the visual and ecological quality of urban environments. *Michelia compressa* is a fragrant, evergreen tree that is highly valued for its flowers and foliage, possessing significant ornamental and economic importance [1]. Introducing this species to higher-latitude regions holds significant importance for the advancement of urban landscaping, as it enriches and enhances the otherwise bleak winter landscapes of northern Chinese cities. *M. compressa* is primarily found in Taiwan, with smaller populations also present in southern Japan, the Ryukyu Islands, Jeju Island in South Korea, and the Philippines [2]. In the early 1980s, the Institute of Botany, Jiangsu Province and Chinese Academy of Sciences (Nanjing Botanical Garden Mem. Sun Yat-Sen) successfully introduced *M. compressa* into Jiangsu Province [3,4]. *M. compressa*, an excellent evergreen ornamental tree belonging to the Magnoliaceae family, exhibits remarkable cold tolerance and represents a promising candidate for greening in the northern subtropics [5].

Cold stress not only restricts the spatial distribution patterns of vegetation but also negatively impacts plant growth and development [6,7]. The rising frequency of unusual climatic events has garnered significant attention to this issue in temperate and cold regions, especially in light of substantial changes in the global climate system [8]. Cold stress can be broadly categorized into two types based on temperature range: low temperature (0–15 °C) and freezing (<0 °C). Low temperatures primarily inhibit various enzymes and cellular processes within metabolic pathways to differing extents, while freezing results in the formation of ice crystals on cell walls, which leads to cellular dehydration [9]. Low temperatures significantly impact plant cell physiology and enzyme activity, resulting in metabolic imbalances and oxidative stress [10,11,12]. Most evergreen *Michelia* species are challenging to cultivate in northern regions in China due to low-temperature limitations. Therefore, understanding the molecular mechanisms of *M. compressa* under cold stress is of significant importance. However, it is noteworthy that the exploration of the stress response in *M. compressa* remains limited, with equally scarce research on gene expression, function, and genetic engineering. However, quantitative real-time PCR (qRT-PCR) has significant limitations, as it can only provide an ‘average’ representation of a mixed population of mRNAs. Each mRNA possesses its own dynamic trajectory, unique stress response, and specific housekeeping genes [13].

Gene expression analysis is a widely employed method for investigating the functional roles of genes that regulate plant growth and development [14,15,16,17]. qRT-PCR technology, known for its rapidity, high precision, high throughput, and sensitivity, is extensively utilized in gene expression analysis research [14]. Several factors, including RNA quality, cDNA quality, sample dilution factor, and experimental accuracy, significantly influence the precision of qRT-PCR [18,19,20]. To minimize these potential sources of error, housekeeping genes (genes that are essential for basic cellular functions) are often utilized as internal reference genes for data standardization. The introduction of appropriate internal reference genes is essential for standardizing the analysis of target gene expression levels. Uncritically adopting reference genes from other experiments without thorough screening can easily lead to inaccurate or even erroneous results. The most commonly used housekeeping genes include actin-related protein (*ACT*), *18S rRNA*, *28S rRNA*, and glyceraldehyde-3-phosphate dehydrogenase (*GAPDH*). It is important to note that housekeeping genes are not infallible; for example, the expression pattern of *18S rRNA* is unstable in potato tubers, while it remains stable in rice [21]. Certain reference genes exhibit stability exclusively in specific tissues or under particular environmental conditions [22]. This observation suggests that housekeeping genes maintain relative stability only within specific tissues or treatment protocols. Therefore, it is essential to select appropriate internal reference genes based on the specific experimental conditions to minimize experimental error.

Auxin response factors (ARFs) are a crucial component of auxin signaling pathways, representing a class of transcription factors that respond to auxin signals [23]. ARFs play a significant role in plant growth and development, as well as in the plant’s responses to abiotic stresses [14,24,25]. Analysis of the *StARF* gene family in potatoes has revealed that most *StARF* genes demonstrate a pronounced response to abiotic stress [26]. Similarly, research in *Arabidopsis thaliana* has shown that the majority of *AtARF* genes are significantly downregulated under conditions of salt and drought stress [27]. In tomatoes subjected to drought stress, the expression levels of the *ARF1*/*4*/*6B*/*10A*/*18* genes in leaves increased markedly [28]. ARF plays a crucial regulatory role in the plant’s response to cold stress by mediating the reprogramming of downstream gene expression, thereby participating in the regulation of cold-induced physiological adaptations [29,30]. While the diversity and complexity of the *ARF* gene family across various species have been extensively studied, research on the functions of *ARF* genes in *M. compressa* remains limited. Therefore, further investigation into the responses of *M. compressa ARF* genes under cold stress is essential. Understanding the biological functions of *ARF* genes in the context of cold stress has significant implications for the breeding of superior cultivars of *M. compressa*.

This study utilized GeNorm, NormFinder, and BestKeeper software to thoroughly evaluate stable internal reference genes in *M. compressa* under cold stress, thereby establishing a robust qRT-PCR detection system. Building on this foundation, bioinformatics methods were employed to identify the *ARF* gene family in *M. compressa* at the full-length transcriptome level. We analyzed the phylogeny, gene structure, motif composition, physicochemical properties, tissue-specific expression patterns, and differential expression under cold stress. One *McoARF* gene was cloned and functionally analyzed, with studies conducted on the responses of transgenic *A. thaliana* to cold stress. These findings establish a molecular foundation for investigating the cold stress response of *M. compressa* and for developing novel germplasm with enhanced stress tolerance.

## 2. Results

### 2.1. Primer Specificity Validation and Amplification Efficiency Analysis for qRT-PCR

**Figure 1:**
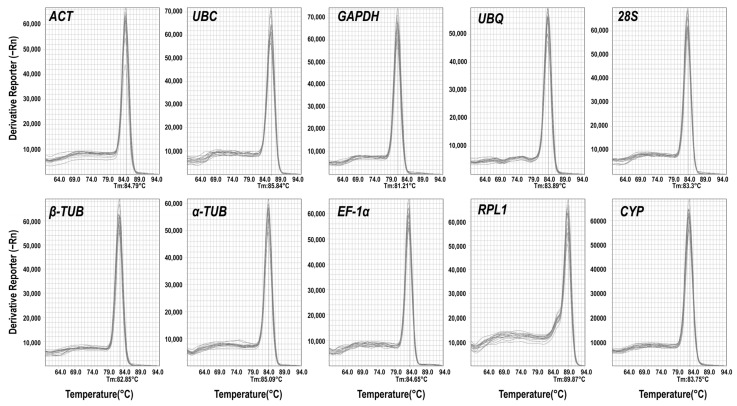
Melting curves of ten candidate reference genes.

Based on the transcriptome data of *M. compressa* (Table S1), we selected *ACT*, *UBC*, *GAPDH*, *UBQ*, *28S*, *β-TUB*, *α-TUB*, *EF-1α*, *RPL1*, and *CYP* as candidate genes for qRT-PCR. PCR amplification was conducted using cDNA that was mixed in equal proportions from all samples as templates. The results demonstrated that all target fragments of the primers were single and distinct, with no evidence of primer dimers or non-specific amplification (Figure S1). The qRT-PCR results showed that the melting curves for each candidate internal reference gene exhibited a single peak, indicating that all primers used achieved specific amplification (Figure 1). Upon calculation, the E for each candidate internal reference gene ranged from 91.53% to 104.35%, while the R^2^ varied from 0.99 to 0.997 (Table 1). These results collectively indicate that each candidate gene possesses good specificity and amplification efficiency, making them suitable for subsequent experiments.

### 2.2. Ct Values of Candidate Reference Genes

**Figure 2:**
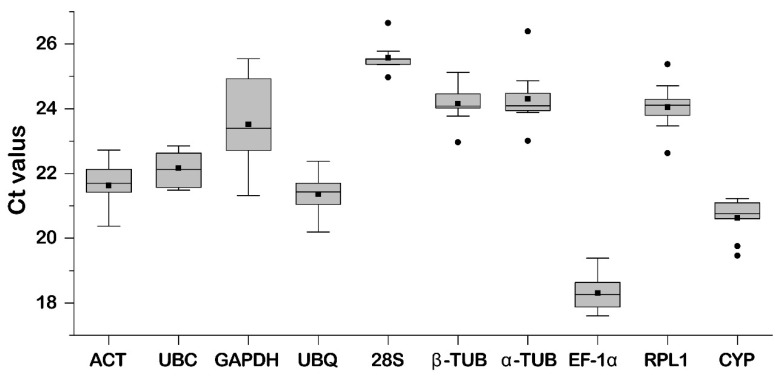
Ct value distribution. The lines in the box represent the median, while the upper and lower boxes indicate the 25th and 75th quantiles, respectively. The points outside the 25th and 75th quantiles represent outlier values.

The Ct value is inversely proportional to gene expression levels; thus, a lower Ct value indicates a higher gene expression level. The experimental results demonstrate that the average Ct value of the ten genes ranges from 18.31 to 25.57, indicating moderate expression abundance (Figure 2). Furthermore, the boxplot analysis suggests that the Ct value of *GAPDH* (21.32~25.54) exhibits a wide range, indicating weak gene expression stability. In contrast, the Ct values of *28S* (24.97~26.64), *β-TUB* (22.96~25.12), and *CYP* (19.46~21.23) show a narrower range, reflecting relatively stable gene expression. These results indicate that different internal reference genes in *M. compressa* exhibit varying expression levels under different treatments. The observed variation in Ct values suggests that *28S*, *β-TUB*, and *CYP* are relatively stable and may serve as the best candidate internal reference genes.

### 2.3. Expression Stability Analysis

**Figure 3:**
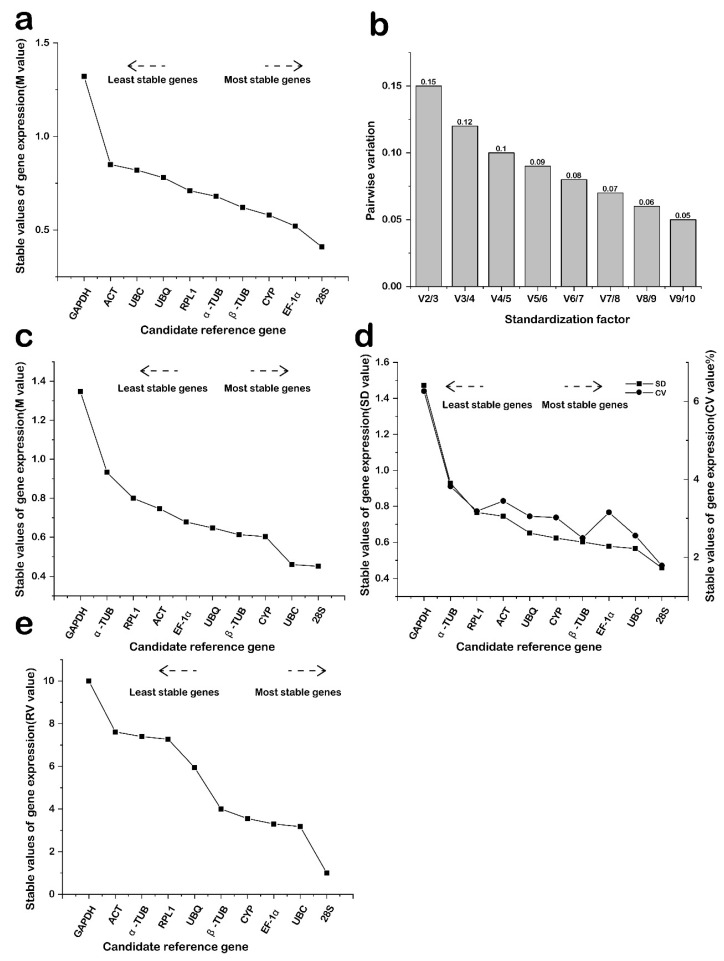
GeNorm, NormFinder and BestKeeper analysis results. (**a**) Average expression stability by GeNorm, (**b**) optimum number, (**c**) average expression stability by NormFinder, (**d**) average expression stability by BestKeeper, (**e**) average expression stability by RefFinder.

The expression stability of each candidate internal reference gene under various treatments and organs during low temperature stress in *M. compressa* was assessed using GeNorm software. The default threshold for the M value is set at 1.5. Based on the M values, the stability of the ten candidate reference genes was ranked. All candidate internal reference genes exhibited M values below 1.5, indicating that their expression is relatively stable (Figure 3a). The stability ranking is as follows: *GAPDH* (1.32) > *ACT* (0.85) > *UBC* (0.82) > *UBQ* (0.78) > *RPL1* (0.71) > *α-TUB* (0.68) > *β-TUB* (0.62) > *CYP* (0.58) > *EF-1α* (0.52) > *28S* (0.41). At the same time, GeNorm can determine the optimal number of reference genes by calculating the pairwise variation value (V_n/n+1_). The critical threshold for V_n/n+1_ is set at 0.15. When V_n/n+1_ is less than 0.15, it indicates that n genes can be selected as internal reference genes, ensuring stable and reliable results. As illustrated in Figure 3b, the value of V_2/3_ is 0.15, while the remaining values are less than 0.15. This suggests that the optimal number of internal parameters for the analysis of *M. compressa* under low-temperature stress should be at least 3.

NormFinder provides stability values for each gene by analyzing Ct values, which serve as direct measures for estimating expression variation when normalized. The expression stability of ten candidate reference genes was further evaluated using the NormFinder algorithm (Figure 3c). The stability ranking is as follows: *GAPDH* (1.35), *α-TUB* (0.93), *RPL1* (0.80), *ACT* (0.75), *EF-1α* (0.68), *UBQ* (0.65), *β-TUB* (0.61), *CYP* (0.60), *UBC* (0.46), and *28S* (0.45). Among these, *28S* was identified as the most stable reference gene across all samples, whereas *GAPDH* exhibited the greatest variation.

Bestkeeper primarily assesses gene stability by comparing the standard deviation (SD) and coefficient of variation (CV) of the Ct values for each candidate intrareal gene. A gene is deemed relatively stable when the SD is less than 1.0. The analysis revealed that, across all treatments and tissues, *28S* was identified as the most stable gene, whereas *GAPDH* was found to be the most unstable gene. These findings are consistent with the results obtained from geNorm and NormFinder (Figure 3d).

The three software programs, geNorm, NormFinder, and BestKeeper, produced incomplete results when evaluating various candidate reference genes, which is inadequate for subsequent gene expression standardization analysis. To further assess the stability of the reference genes, the analysis results obtained from geNorm, NormFinder, and BestKeeper were organized according to the comprehensive rankings derived from all three software applications (Figure 3e). The comprehensive stability rankings of each gene, ordered from highest to lowest, are as follows: *GAPDH* (10), *ACT* (7.61), *α-TUB* (7.40), *RPL1* (7.27), *UBQ* (5.94), *β-TUB* (4), *CYP* (3.56), *EF-1α* (3.30), *UBC* (3.17), and *28S* (1). This indicates that *28S* exhibits greater expression stability compared to the other genes and can be utilized as the primary internal reference gene for qRT-PCR analysis of *M. compressa* under low temperature stress. Conversely, the expression of the *GAPDH* (10) gene is the most unstable and should not be employed as the internal reference gene for *M. compressa* under low temperature stress.

### 2.4. Gene Family Member Identification and Protein Physicochemical Properties Analysis

Based on the full-length transcriptome data of *M. compressa*, a total of 48 *McoARF* members were identified and designated as *McoARF1* to *McoARF48*. The physicochemical characteristics of the *McoARF* genes and their corresponding proteins were analyzed (Table S4). The encoded amino acid lengths ranged from 586~1533 aa, with molecular weights varying from 65.93~173.02 kD. The isoelectric points ranged from 5.74~9.93, indicating that most *McoARFs* are composed of acidic amino acids, suggesting their primary function in a weakly acidic cellular environment. Furthermore, predictions regarding subcellular localization indicated that all 48 members of the *ARF* family are localized in the nucleus.

### 2.5. Phylogenetic Analysis of Gene Family

**Figure 4:**
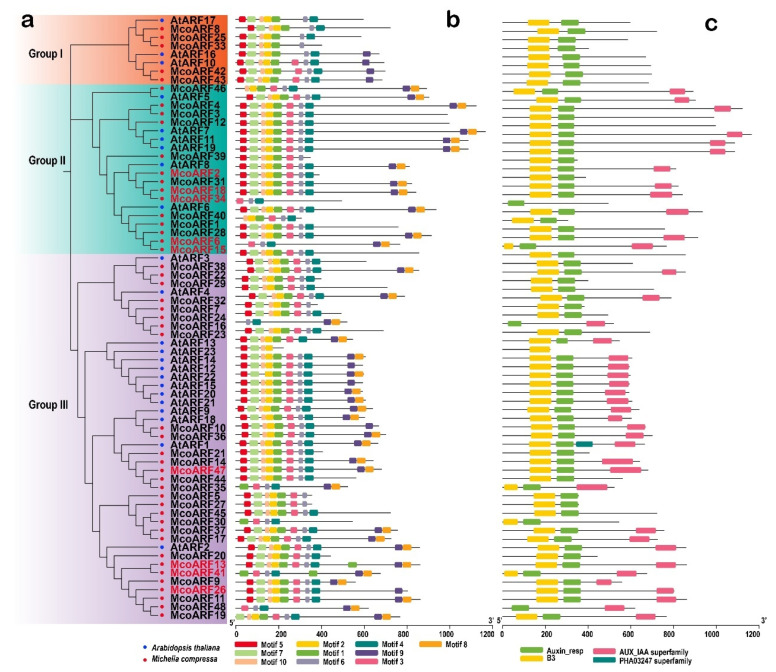
Conserved motifs and conserved domain of ARF proteins in *M. compressa*. (**a**) Evolutionary relationship, (**b**) conserved motifs, (**c**) conserved domain.

To elucidate the evolutionary relationship and classification of the *M. compressa* ARFs protein, this study utilized 23 *A. thaliana* ARFs protein sequences for comparative analysis with McoARFSs protein sequences, leading to the construction of a phylogenetic tree (Figure 4a). Based on the classification results, the 48 McoARFSs proteins were categorized into three subfamilies: 5 members in Group I, 14 members in Group II, and 29 members in Group III.

### 2.6. Structural and Conserved Motif Analysis

To understand the conservative structure and evolutionary relationship of McoARF proteins, this study analyzed the motif composition of 48 McoARFs proteins using the online MEME program, resulting in the identification of 10 distinct motifs (Motifs 1–10) (Figure 4b and Figure S2). The majority of McoARF proteins exhibit motifs 5, 7, 10, and 2, indicating that these motifs are crucial components of the McoARF protein family. Notably, McoARF34 is unique in containing only motif 3, 6 and 4. Furthermore, most McoARFs proteins possess both the Auxin_resp and B3 structures, with one McoARF protein containing the B3 structure (Figure 4c). Specific motifs contribute to the formation of distinct domains; the Auxin_resp domain is primarily composed of motifs 1, the B3 domain is primarily composed of motifs 3, 6 and 4, while the AUX_IAA domain is predominantly composed of motifs 8 and 9.

### 2.7. Expression of McoARF Genes Under Cold Stresses

**Figure 5:**
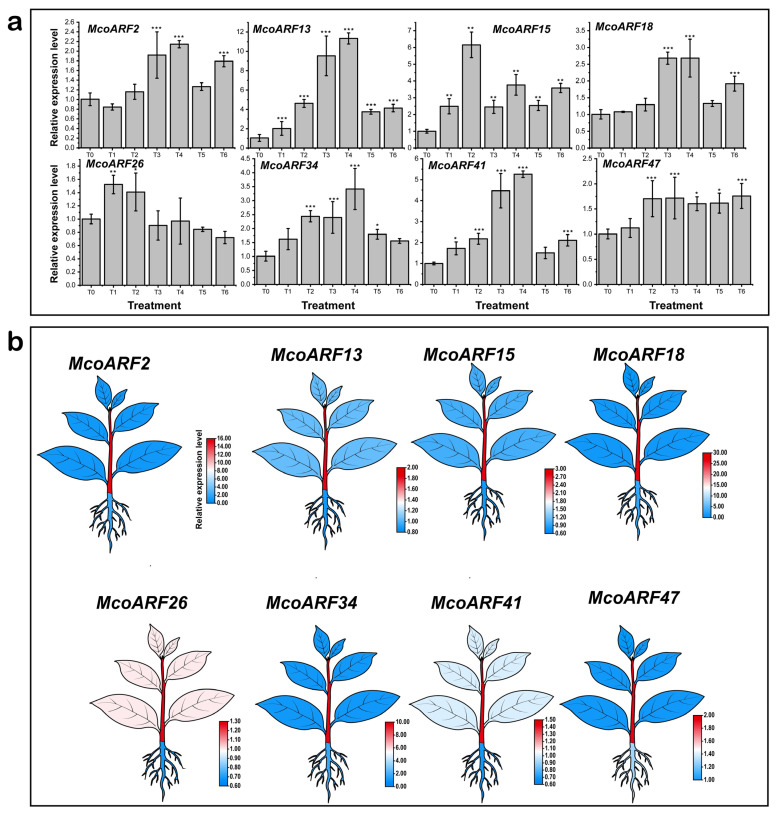
Expression analysis of *McoARF* genes. (**a**) *McoARF* genes expression in leaves under cold stress, (**b**) leaves, stems, and roots. *: Significant difference at *p* < 0.05; **: Very significant difference at *p* < 0.01; ***: Extremely significant difference at *p* < 0.001.

To further analyze the expression patterns of *McoARF* genes in *M. compressa* under cold stress, qRT-PCR technology was utilized to examine the expression profiles of eight *ARF* family genes across various cold stress treatments and their expression characteristics in different organs. The genes analyzed included *McoARF2*, *McoARF13*, *McoARF15*, *McoARF18*, *McoARF26*, *McoARF34*, *McoARF41*, and *McoARF47*. Results indicate that the relative qRT-PCR expression levels of most genes in leaves increased with prolonged cold stress duration, with the majority reaching peak expression at 36 or 48 h (Figure 5a). Notably, four genes *MoARF13*, *McoARF15*, *McoARF26*, and *McoARF41* demonstrated significantly higher relative qRT-PCR expression levels at 12 h of cold stress compared to the untreated control group. Furthermore, the relative qRT-PCR expression levels of *McoARF13* and *McoARF15* were significantly elevated in all six cold-stressed treatments (12, 24, 36, 48, 60, and 72 h) compared to the unstressed control. Among these, the expression of *McoARF13* exhibited an initial increase followed by a decrease. The expression patterns of the nine genes across the three organs (leaf, stem, and root) revealed that the relative qRT-PCR expression levels in stems were significantly higher than those in leaves and roots (Figure 5b). This finding suggests that the response patterns of different *McoARF* genes exhibit temporal and expression pattern differences, implying that they may possess distinct functions and regulatory mechanisms in cold regulation. Therefore, *McoARF13* was selected for subsequent functional validation.

### 2.8. Phenotypic Validation of McoARF13 Transgenic A. thaliana

This study utilized cDNA from *M. compressa* leaves as a template, designing specific primers based on transcriptome sequences to clone the *McoARF13* gene, which has an ORF of 2581 bp (Figure S3a). The protein’s secondary structure predominantly consists of disordered coils (74.39%), with α-helices accounting for 14.67% and extended chains comprising approximately 10.94% (Figure S3b). Three-dimensional structure prediction of the *McoARF13* protein revealed a conformation consistent with the secondary structure analysis, again dominated by disordered coils (Figure S3c). The prediction of phosphorylation sites in the *McoARF13* protein identified 96 phospho-modification sites: 60 serine sites, 30 threonine sites, and 6 tyrosine sites (Figure S3d).

**Figure 6:**
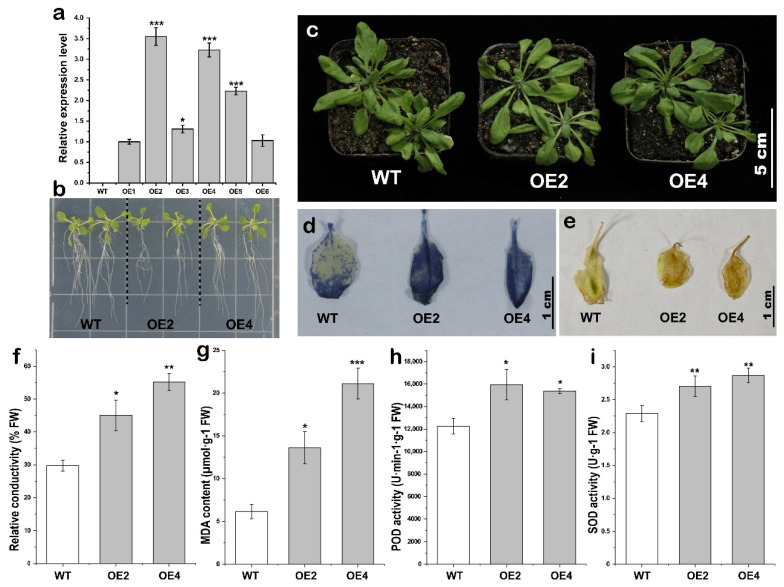
Overexpression of *McoARF13* reduced the tolerance of transgenic *A. thaliana* to cold stress. (**a**) Analysis of the expression of transgenic *A. thaliana* using qRT-PCR, (**b**) T3 transgenic *A. thaliana* seedlings and WT seedlings, (**c**) cold stress phenotype in *A. thaliana*, (**d**) NBT staining of leaves after cold stress, (**e**) DAB staining of leaves after cold stress, (**f**) relative conductivity, (**g**) MDA content, (**h**) POD activity, (**i**) SOD activity. *: Significant difference at *p* < 0.05; **: Very significant difference at *p* < 0.01; ***: Extremely significant difference at *p* < 0.001.

To investigate the effect of *McoARF13* on plant cold stress response, this study employed the inflorescence infection method to screen for homozygous T3 transgenic *A. thaliana* lines using kanamycin for subsequent experimental analysis. RT-PCR confirmed the expression of the target gene in both the vector plasmid and all overexpression (OE) plants, while no band was detected in the wild-type (WT) plants (Figure S4). Subsequently, qRT-PCR analysis was conducted to assess the expression levels of the target gene, revealing no expression in the WT strain. Given the higher expression levels observed in the *McoARF13*-OE2 and *McoARF13*-OE4 lines, these two independent transgenic lines were selected for further analysis (Figure 6a). Under normal growth conditions, no significant difference in growth was observed between the WT and *McoARF13*-OE *A. thaliana* lines (Figure 6b). However, after 24 h of cold stress at 4 °C, *McoARF13*-OE seedlings exhibited significantly greater wilting compared to the WT (Figure 6c). This study analyzes the physiological and biochemical responses of *A. thaliana* following cold stress as key indicators of cold stress response. Nitroblue tetrazolium chloride (NBT) staining revealed that after 24 h of stress, a greater proportion of leaves in the *McoARF13*-OE2 and *McoARF13*-OE4 lines exhibited blue coloration, indicating an increased accumulation of reactive oxygen species (ROS) in the leaves (Figure 6d). Conversely, 3,3′-diaminobenzidine (DAB) staining revealed that after 24 h of stress, the WT leaves exhibited fewer brown spots compared to the *McoARF13*-OE2 and *McoARF13*-OE4 (Figure 6e). Measurements of relative electrical conductivity and malondialdehyde (MDA) content in *A. thaliana* following stress exposure indicated that both *McoARF13*-OE2 and *McoARF13*-OE4 lines exhibited significantly higher levels than WT. This suggests that *McoARF13*-OE2 and *McoARF13*-OE4, which possess lower cold stress response, accumulated greater amounts of osmotic substances in response to cold stress (Figure 6f,g). Furthermore, changes in peroxidase (POD) and superoxide dismutase (SOD) activity were correlated with osmotic solute levels. This suggests that the severe stress experienced by the *McoARF13*-OE2 and *McoARF13*-OE4 lines following cold exposure resulted in increased POD and SOD activity as a defense mechanism against stress-induced damage. (Figure 6h,i). Collectively, these findings indicate that the overexpression of *McoARF13* markedly reduces the cold stress response of transgenic *A. thaliana* under cold stress.

## 3. Discussion

Cold stress significantly constrains tree growth. Investigating the stress resistance mechanisms of *M. compressa*, deciphering its gene expression regulatory network in response to cold stress, and identifying relevant stress-related genes will enhance our understanding of the cold stress adaptation mechanisms in *M. compressa*. qRT-PCR is one of the primary methods for analyzing gene expression levels and regulatory patterns, and it is widely employed for detecting gene expression levels [12,31,32]. However, qRT-PCR effectively measures the average gene expression across all cell types. This homogenization of the tissue obscures the specific responses of different cell types to stress, leading to the molecular changes occurring within each cell type being ‘diluted’ [33]. In non-model plants, combining plant transcriptome databases for the selection of internal reference genes is an effective approach [18]. Previous research indicates that housekeeping genes lack absolute universality; their selection outcomes are not consistently stable across different plant tissues and experimental conditions [34]. Employing commonly used housekeeping genes as internal controls without prior screening significantly diminishes the accuracy of results and may even yield erroneous outcomes. Past gene expression studies in *Michelia* frequently utilized GAPDH as a direct internal control, which could potentially introduce considerable error [35]. In recent years, advancements in modern molecular biology research have established a crucial foundation for subsequent functional gene expression analyses through the screening of housekeeping genes in *M. compressa*. Consequently, to standardize data processing, it is essential to select appropriate housekeeping genes before analyzing the expression levels of target genes.

Using the three analysis software packages, geNorm, NormFinder, and BestKeeper, suitable housekeeping genes have been identified across multiple species, including *Magnolia* × *soulangeana* [20], *Piper* [19], and tree peony [36], etc. However, inconsistent results among the analyses conducted by geNorm, NormFinder, and BestKeeper are frequently observed across different species [37]. Research has revealed that the expression stability of the housekeeping genes *TIP41* and *CYP*, identified through transcriptome database mining of *Melaleuca bracteata*, surpasses that of the conventional housekeeping gene *ACT* [38]. Differences in the results produced by GeNorm, NormFinder, and BestKeeper software are common across species [19,34,36,37]. This finding indicates that a comprehensive assessment of gene stability, utilizing multiple distinct algorithms to screen candidate housekeeping genes, yields accurate and reliable results.

As a key transcription factor within the auxin signaling pathway, ARF regulates the expression of downstream target genes [39,40]. Research has demonstrated that the ARF family is crucial for plant growth, development, hormone responses, and stress responses, establishing it as one of the essential gene families for understanding plant biology [14,24,41]. The ARF gene was initially identified as comprising 23 members in the model plant *A. thaliana* and it has since been identified in numerous other plants [23]. For example, *colored potatos* has 55 *ARF* members [42], rice has 25 *ARF* members [16], *Pinus koraiensis* has 13 *ARF* members [43], *Betula platyphylla* has 15 *ARF* members [24], *Medicago sativa* has 81 *ARF* members [14], etc. This study identified 48 members of the *McoARFs* gene family from the full-length transcriptome sequences of *M. compressa*. The moderate number of *ARF* family members in *M. compressa* indicates significant variability in *ARF* gene family size across different plant species. Given that the complete genome sequence of *M. compressa* remains unpublished, the reliance on full-length transcriptomes presents certain limitations; consequently, the final count of *ARF* members in *M. compressa* may increase. The prediction of subcellular localization indicates that all 48 members of the *McoARFs* family are localized within the cell nucleus, suggesting that McoARFs primarily function as transcription factors in this compartment. This observation is consistent with the subcellular localization findings reported for most ARF family proteins in other species, indicating that the subcellular localization of ARF proteins is relatively conserved across different organisms.

The *ARF* gene family plays a significant role in plant responses to abiotic stress, facilitating the maintenance of normal growth and development under adverse conditions by regulating auxin signaling, cytokinin levels, and other hormonal pathways [14,24]. Notably, ARF genes show substantial changes in expression in response to various stress conditions, which are essential for plant survival and adaptation. This research will employ full-length transcriptome data alongside a novel bioinformatics approach to identify *McoARF* genes associated with cold stress response and regulation in *M. compressa*. The study analyzed the expression patterns of nine *McoARF* genes under cold stress conditions. All nine *McoARF* genes displayed distinct expression trends in reaction to cold stress. It is noteworthy that both *McoARF13* and *McoARF18* exhibited significantly elevated expression levels following all six cold stress treatments in *M. compressa*. This observation indicates that these genes play crucial roles in the *M. compressa* response to cold stress. Previous studies have demonstrated that *GmARF16* plays a pivotal role in the salt stress response of soybean, establishing a gene regulatory network with *miR160a* and *GmMYC2* [44]. Additionally, research on *B. platyphylla* has shown that the expression of *BpARF1* is significantly induced by drought stress [24]. Furthermore, functional analyses of *Cucurbita pepo* under drought and salt stress conditions have indicated that the overexpression of *CpARF22* in *A. thaliana* markedly enhances both salt tolerance and drought resistance [25]. Notably, the expression levels of these nine *McoARF* were significantly higher in the stems of *M. compressa* compared to the leaves and roots, suggesting that these transcription factors may have tissue-specific functions. *ARF* genes play a significant role in stems, which aligns with the expression patterns of ten *HsARF* genes in *Huperzia serrata* [45]. Previous studies have reported that *ARF* genes are involved in temperature responses in various plant species. For instance, in Sorghum bicolor, the genes *SbARF6/24/25* are significantly induced by cold stress, while *SbARF16/22* are activated by heat stress [29]. Similarly, in rice, *OsARF11/13/16* respond to cold stress, whereas *OsARF4/14/18/19* are induced by heat stress [46]. Chen et al.’s [14] research on cold stress in *Medicago sativa* demonstrated that the majority of genes showed upregulation exclusively at the 12 h time point within certain tissues. This finding indicates a significant correlation between the regulation of these genes and the duration of the cold-stress event. These findings further underscore the critical role of *ARF* genes in the plant response to temperature stress.

As a family of plant-specific TF, ARFs act cell-type-specifically and strictly as a cascade; they have emerged as crucial regulators in response to various abiotic stresses. Increasing evidence indicates that *ARF* family genes play a significant role in plant responses to these stresses [14,25,39]. Numerous studies demonstrate that *ARF* genes predominantly exert positive regulation on plant stress resistance. Moreover, certain studies on *ARFs* have demonstrated their negative regulation of plant stress resistance. For instance, research on soybean salt tolerance revealed that soybean plants overexpressing *GmARF16* exhibited significantly reduced salt tolerance, while *GmARF16* RNA interference (RNAi) markedly enhanced the salt tolerance of soybean plants [44]. Research indicates that in plants where *BpARF1* RNAi has been suppressed, *B. platyphylla* demonstrates a reduction in ROS accumulation, which subsequently enhances the tree’s drought tolerance [24]. In this study, the overexpression of the *McoARF13* gene in *A. thaliana* significantly diminished cold stress tolerance following cold treatment, thereby supporting its role in negatively regulating the cold stress response.

**Figure 7:**
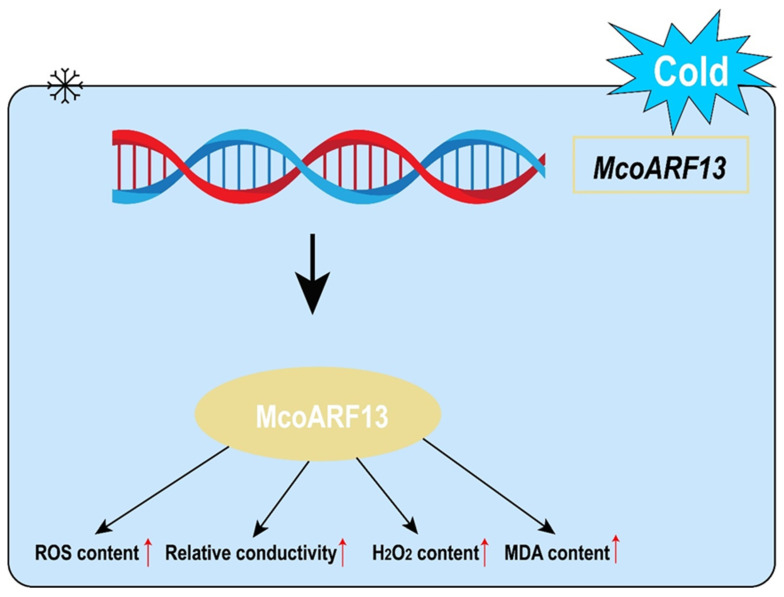
Schematic diagram of the mechanism by which *McoARF13* responds to cold stress in leaves. The arrow (↑) denotes a stimulatory or inducing effect of McoARF13 on the downstream parameters.

When plants experience abiotic stress, significant amounts of free radicals and ROS accumulate within the plant tissue, middle increasing level. Concurrently, the levels of certain enzymes and non-enzymatic substances that scavenge free radicals and ROS increase, thereby regulating membrane permeability and enhancing both the structural and functional stability of membranes. This process serves to protect plant cells from damage. In *Cuphea hookeriana* subjected to cold stress, the contents of MDA, soluble proteins, and free proline progressively increased with the duration of treatment [12]. In contrast, the activities of SOD and catalase (CAT) enzymes initially rose before subsequently declining [12]. Furthermore, studies on cold stress in tea plants revealed that overexpression of *CsFKBP53* in *A. thaliana* led to significantly enhanced activities of POD, SOD, and CAT in the OE plants compared to the WT plants [47]. In this study, the overexpression of *McoARF13* in *A. thaliana* was found to reduce tolerance to cold stress. This was evidenced by significant increases in ROS, hydrogen peroxide (H_2_O_2_), and MDA levels, along with elevated activities of POD and SOD enzymes. The increase in ROS and MDA levels indicates more severe damage to cell membranes following *McoARF13* overexpression. The notable rise in POD and SOD enzyme activities may reflect an initial response to cold stress, where the rapid activation of these enzymes enhances the plant’s adaptation to cold conditions. However, as substances regulating osmotic pressure during emergency responses, the activities of SOD and POD ultimately decline over time. These findings further indicate that *McoARF13* plays a critical role in maintaining ROS homeostasis by negatively regulating the antioxidant system (Figure 7). This study establishes a foundational basis for exploring the molecular mechanisms through which ARF TF genes confer stress resistance in *M. compressa*, and it further elucidates the stress-tolerance functions of members within the woody plant ARF TF gene family.

## 4. Materials and Methods

### 4.1. Plant Materials

Two-year-old *M. compressa* seedlings were planted in flowerpots measuring 10 cm × 10 cm × 8.5 cm, filled with a substrate mixture of peat, yellow mud, and perlite in a volume ratio of 2:2:1. The experimental materials were placed in the nursery of the Institute of Botany, Jiangsu Province and China Academy of Sciences (32°3′29″ N, 118°49′56″ E). Following management protocols, the plants were watered regularly and, after one month of survival, were moved to an artificial climate culture room maintained at a temperature of 22–25 °C, with a light intensity of 0.1 mmol/m^2^·s, a light cycle of 16 h, and humidity set at 80%. After four weeks of incubation, samples of leaves, stems, and roots were collected prior to low-temperature treatment. Subsequently, the plant materials were transferred to an artificial climate chamber at 4 °C, while other conditions remained unchanged. Leaves were harvested at 0 (T0), 12 (T1), 24 (T2), 36 (T3), 48 (T4), 60 (T5), and 72 h (T6) after treatment. All samples were collected from fully developed mature leaves that grew in the experimental year. The leaves were frozen in liquid nitrogen and stored at −80 °C, with three replicates taken from each sample.

### 4.2. RNA Extraction and cDNA Synthesis

Total RNA was extracted using the FastPure Universal Plant Total RNA Isolation Kit (Vazyme, Nanjing, China) following the manufacturer’s instructions. RNA concentration was measured with a spectrophotometer (NanoDrop2000, Thermo Scientific, Waltham, MA, USA), with a threshold standard of 1.8 to 2.2 at 260/280 nm. cDNA synthesis was conducted with the Evo M-MLV RT Kit (with gDNA Clean for qPCR) (Accurate, Changsha, China), adhering to the product manual and utilizing 1 μg of RNA for the synthesis of the first strand cDNA each time.

### 4.3. Primer Design of Reference Genes

According to the transcriptome sequence of *M. compressa*, ten candidate internal reference genes were selected, including actin-related protein (*ACT*), ubiquitin conjugating enzyme (*UBC*), glyceraldehyde-3-phosphate dehydrogenase (*GAPDH*), ubiquitin protein (*UBQ*), *18S rRNA*, *28S rRNA*, tubulin beta (*β-TUB*), tubulin alpha (*α-TUB*), elongation factor 1 alpha (*EF-1α*), large subunit ribosomal protein L1 (*RPL1*), and cyclophilin (*CYP*) (Table S1). Primers were designed using the National Center for Biotechnology Information (NCBI) website, and Table 1 provides the primer sequences, amplification product lengths, and Tm values. The primers were synthesized by Generay (Shanghai, China). cDNA samples were diluted into five gradients and amplified using qRT-PCR. Finally, standard curves for the internal reference genes were generated based on the amplification results, and the correlation coefficient (R^2^) and amplification efficiency (E) were calculated. Additionally, the specificity of the PCR products was assessed through 1% agarose gel electrophoresis.

**Table 1:** Primer sequences and amplification efficiency of 10 candidate reference genes in *M. compressa*.

Gene	Primer Sequence (5′→3′)	Product Length (bp)	E%	R^2^
*ACT*	F: TGTTAGCCACACAGTGCCAA R: GCTCTTTTCAACAGCGGAGC	244	96.20	0.991
*UBC*	F: GCGCCATCTCTGAGTCTACC R: CAGTGGCAACAACAGACAGC	222	104.35	0.994
*GAPDH*	F: CGGGTCTTCCGTTATCTCCG R: CCAACCTTCCAATCCGACCA	170	91.53	0.990
*UBQ*	F: GAGGATGGTCGTACGCTTGC R: AGGGTCCTTCCATCTTCCAAC	245	99.15	0.995
*28S*	F: GCAGCACAAGCGTGTACAAA R: CCGTGGCACTGTATGGAACT	177	96.70	0.997
*β-TUB*	F: CATTGCCAGCCCCAAAACTC R: AGGCTGTTTGGATGGAGAGC	170	96.64	0.990
*α-TUB*	F: TCATTCATGCGGCTGGAACT R: CTTATTAGCCCCGCACCCAT	231	99.14	0.995
*EF-1α*	F: TCATCATGAACCACCCAGGC R: CACGGCAAAACGTCCAAGAG	245	94.15	0.993
*RPL1*	F: TTGCTAAGGAGACGGCCAAG R: GCATAACCCGAGCGATCTCA	216	95.34	0.992
*CYP*	F: CCAGATACGAATGGGTCGCA R: CTGCAATCACCACTTTCCGC	166	97.64	0.995

### 4.4. qRT-PCR

qRT-PCR was conducted utilizing the SYBR Green dye method. The PCR reaction mixture was prepared in accordance with the instructions provided by the 2 × SYBR Green Premix Pro Taq HS qPCR Kit (Accurate, Changsha, China), comprising the following components: cDNA template (2 μL), forward and reverse primers (10 μM) at 0.4 μL each, 2 × SYBR Green Premix Pro Taq HS qPCR Kit (10 μL), and the total volume adjusted to 20 μL with ddH_2_O. Amplification was performed using a StepOnePlus real-time PCR system (Applied Biosystems, Waltham, MA, USA) under the following conditions: an initial denaturation step of 10 min at 95 °C, followed by 40 cycles of 15 s at 95 °C, 30 s at 60 °C, and 30 s at 72 °C, concluding with a melting curve analysis from 60 °C to 95 °C. Each sample was analyzed in triplicate.

### 4.5. Data Analysis of Gene Expression Stability

Ten candidate internal reference genes were comprehensively evaluated using three gene stability evaluation programs (GeNorm, NormFinder, BestKeeper) and the online analysis tool RefFinder to identify the most suitable internal reference gene for *M. compressa* [43,48,49,50]. In the analysis of the stability of internal reference gene expression, raw Ct values are transformed into 2^−ΔCt^ when using geNorm and NormFinder. Conversely, BestKeeper utilizes the raw Ct values to compute the coefficient of variation (CV) and standard deviation (SD) for the expression of candidate internal reference genes. Additionally, geNorm employs 2^−ΔCt^ as the raw data to ascertain the optimal number of internal reference genes by calculating the paired difference values V_n_/V_n+1_ of two consecutive normalized factors.

### 4.6. Bioinformatics Analysis

#### 4.6.1. Identification and the Physicochemical Attributes of McoARF Members

Based on the full-length transcriptome sequencing data of *M. compressa*, two methods were employed to identify *McoARFs*: First, protein sequences from the *AtARF* gene family served as seed sequences for a BLAST alignment, which was conducted against the full-length transcriptome database of *M. compressa* with a screening threshold of E ≤ 10^−5^. Second, the HMM search module of TBtools was utilized to search the full-length transcriptome database for redundant Unigene protein sequences using the hidden Markov model of PF06507. The candidate proteins identified through these two methods were combined, after which repeated sequences were eliminated, and redundant or mispredicted genes were manually removed. The final sequences were designated as *McoARF1* to *McoARF48*. The basic physicochemical properties of *McoARFs* were analyzed using TBtools, while WOLF (https://wolfpsort.hgc.jp/, accessed on 24 January 2026) was employed for subcellular localization prediction analysis [51].

#### 4.6.2. Phylogenetic Analysis of ARF Genes

The *ARF* gene family protein sequences of *A. thaliana* were downloaded from the Phytotome website (https://phytozome-next.jgi.doe.gov/, accessed on 24 January 2026) [23]. The amino acid sequences of *A. thaliana* and *M. compressa* were aligned using MEGA 12 software, and phylogenetic trees were constructed employing the Neighbor-Joining method [52]. The evolutionary tree was enhanced using the Chiplot online tool (https://www.chiplot.online/, accessed on 24 January 2026).

#### 4.6.3. Analysis of Conserved Motifs in ARF Genes

The NCBI-CDD online tool was utilized to predict the conserved domains of *McoARFs* (https://www.ncbi.nlm.nih.gov/cdd, accessed on 24 January 2026). Conserved motifs of *McoARFs* were identified using the MEME 5.5.9 software (https://meme-suite.org/meme/tools/meme, accessed on 24 January 2026) and visualized with TBtools software.

#### 4.6.4. Bioinformatics and Expression Analysis of M. compressa McoARF13 Gene

Utilizing BioXM software (version 2.7.1), we analyzed the amino acid composition of *McoARF13*. The SOPMA website (https://npsa.lyon.inserm.fr/cgi-bin/npsa_automat.pl?page=/NPSA/npsa_sopma.html, accessed on 24 January 2026) was employed for predicting the protein’s secondary structure, while Swiss-Model (https://swissmodel.expasy.org/) was used for tertiary structure prediction. Additionally, phosphorylation sites were analyzed using NetPhos 3.1 (https://services.healthtech.dtu.dk/services/NetPhos-3.1/, accessed on 24 January 2026).

### 4.7. Gene Expression Analysis

Based on the transcriptomic data of *M. compressa*, eight *ARF* genes were randomly selected to examine their expression patterns across various tissues and under different treatments. The primer design and synthesis procedures were consistent with those described in Section 2.3, and the primer sequences are provided in Table S2. The results of the qRT-PCR were analyzed using the 2^−ΔΔCt^ method [53].

### 4.8. Gene Cloning and Sequence Alignment

Based on the transcript sequence obtained from transcriptome sequencing, the coding sequence (CDS) of the target gene *McoARF13* was identified using the Open Reading Frame (ORF) Finder. Recombinant arm primers were designed to incorporate restriction sites (XbaI and BamHI) using the Vazyme online tool (https://tool.vazyme.com:18002/cetool/simple.html, accessed on 28 January 2026). PCR amplification was conducted using Phanta Flash Super-Fidelity DNA Polymerase (Vazyme, Nanjing, China) (primers were listed in Table S3). Subsequently, the *McoARF13* sequence was inserted into the pBI121 vector using the ClonExpress II One Step Cloning Kit (Vazyme, Nanjing, China). The recombinant vector was then transformed into *Escherichia coli* DH5α (Coolaber, Beijing, China), followed by PCR validation of the bacterial culture. Sequencing was conducted, and the resulting sequences were aligned using BioXM software (version 2.7.1).

### 4.9. Construction, Purification, and Stress Treatments for Transgenic Arabidopsis

The 35S:*McoARF13* plasmid was transformed into *Agrobacterium tumefaciens* GV3101 (Coolaber, Beijing, China), followed by the infection of *A. thaliana* Col-0 plants using the inflorescence method [54]. The plants were incubated in darkness for 24 h. Seeds were harvested and screened on 1/2 MS (Coolaber, Beijing, China) medium containing 50 mg·L^−1^ kanamycin (Coolaber, Beijing, China). Positive candidates were identified through PCR and qRT-PCR, with *AtACT* serving as the internal control gene (primers are listed in Table S3). T3 transgenic *A. thaliana* was utilized for gene functional validation. One-month-old T3 and WT plants were subjected to cold treatment at 4 °C for 24 h. The leaves were immersed in a 10 mM potassium phosphate buffer containing 0.5 mg·mL^−1^ NBT and incubated in the dark for 3 h [54]. Following decolourisation with ethanol, the distribution of ROS was observed. To visualise the location of H_2_O_2_, the leaves were immersed in a 1 mg·mL^−1^ aqueous solution of DAB and treated for 12 h, followed by decolourisation with ethanol before observation [54]. Relative electrical conductivity was measured using the immersion method; MDA was determined by the thiobarbituric acid colorimetric assay; POD activity was assessed using the guaiacol method; and SOD activity was quantified by the nitroblue tetrazolium reduction assay [12,54].

### 4.10. Statistical Analysis

The Origin 2021 software was utilized for drawing charts. We utilized IBM SPSS Statistics 27 to conduct a one-way analysis of variance (ANOVA) in order to evaluate the significance of differences between the treatment group and the control group (* *p* < 0.05, ** *p* < 0.01, *** *p* < 0.001). Images were merged using Adobe Photoshop CC 2017 and Adobe Photoshop CS5.

## 5. Conclusions

This study presents the first systematic evaluation of ten candidate housekeeping genes for normalizing qRT-PCR gene expression data in *M. compressa* under cold stress across three distinct organs. The expression stability of these ten candidate genes was evaluated using GeNorm, NormFinder, and BestKeeper. This study identifies the *28S* gene as the optimal housekeeping gene for qRT-PCR detection of cold stress events in *M. compressa*. The *ARF* gene family was characterized through a comprehensive full-length transcriptome analysis, which included predictions of essential physicochemical properties. The research investigated the phylogenetic relationships, gene structures, and expression patterns of *ARF* genes in response to cold stress and across various tissues. A total of 48 *McoARF* genes were identified and categorized into three distinct groups. The relative expression levels of nine *McoARFs* were generally found to be elevated under cold stress conditions. Furthermore, the overexpression of *McoARF13* in *A. thaliana* resulted in a reduced cold stress response in the plants. In summary, these findings establish a theoretical foundation for further elucidating the role of *McoARFs* in the cold stress response of *M. compressa*.

## Data Availability

The raw PacBio and RNA-seq data have been submitted to the Genome Sequence Archive (GSA, https://ngdc.cncb.ac.cn/gsub/submit/gsa/, accessed on 1 January 2026) under accession numbers CRA031930 and CRA031943.

## Supplementary Materials

The following supporting information can be downloaded at: https://www.mdpi.com/article/10.3390/plants15091317/s1, Figure S1: Agarose gel electrophoresis of conventional PCR products of candidate reference genes of *M. compressa*; Figure S2: Investigating motif information in amino acid sequences; Figure S3: *McoARF13* cloning and bioinformatics analysis; Figure S4: PCR detection of the expression of the positive plant *McoARF13*; Table S1: Housekeeping gene candidate read count, FPKM, and annotation; Table S2: Primer sequences for qRT-PCR in *McoARF*; Table S3: Primers for functional validation of *McoARF13*; Table S4: Member information and protein physicochemical properties of the *ARF* gene family in *M. compressa*.
