# Supplementary material for: Investigation of Reference Genes for qRT-PCR and ARF Gene Family in Michelia compressa (Magnoliaceae) Under Cold Stress
Source: Plants (Basel). 2026 Apr 25;15(9):1317. doi: 10.3390/plants15091317 (PMC13164995; doi:10.3390/plants15091317)
Supplement: Supplementary file 1 [file plants-15-01317-s001.zip › Supplementary file S2.pdf]

Supplementary figure

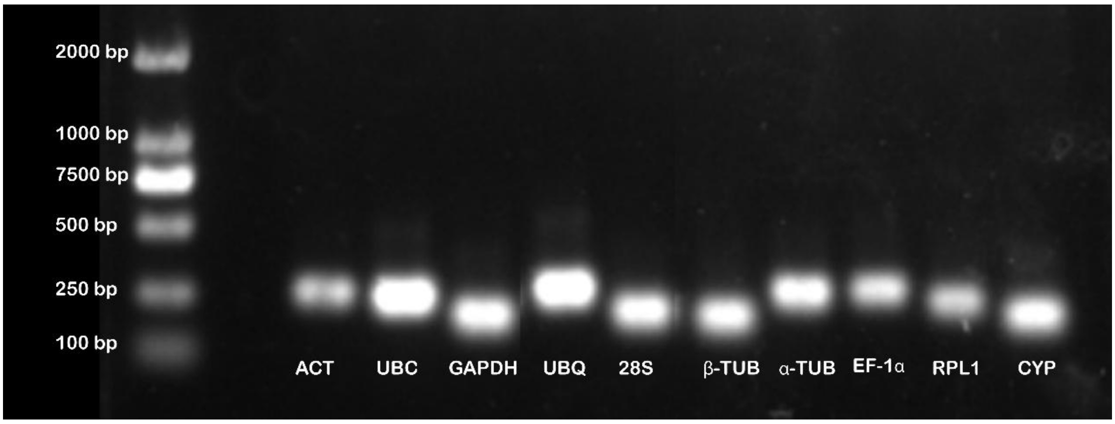

Figure S1. Agarose gel electrophoresis of conventional PCR products of candidate reference genes of *M. compressa*

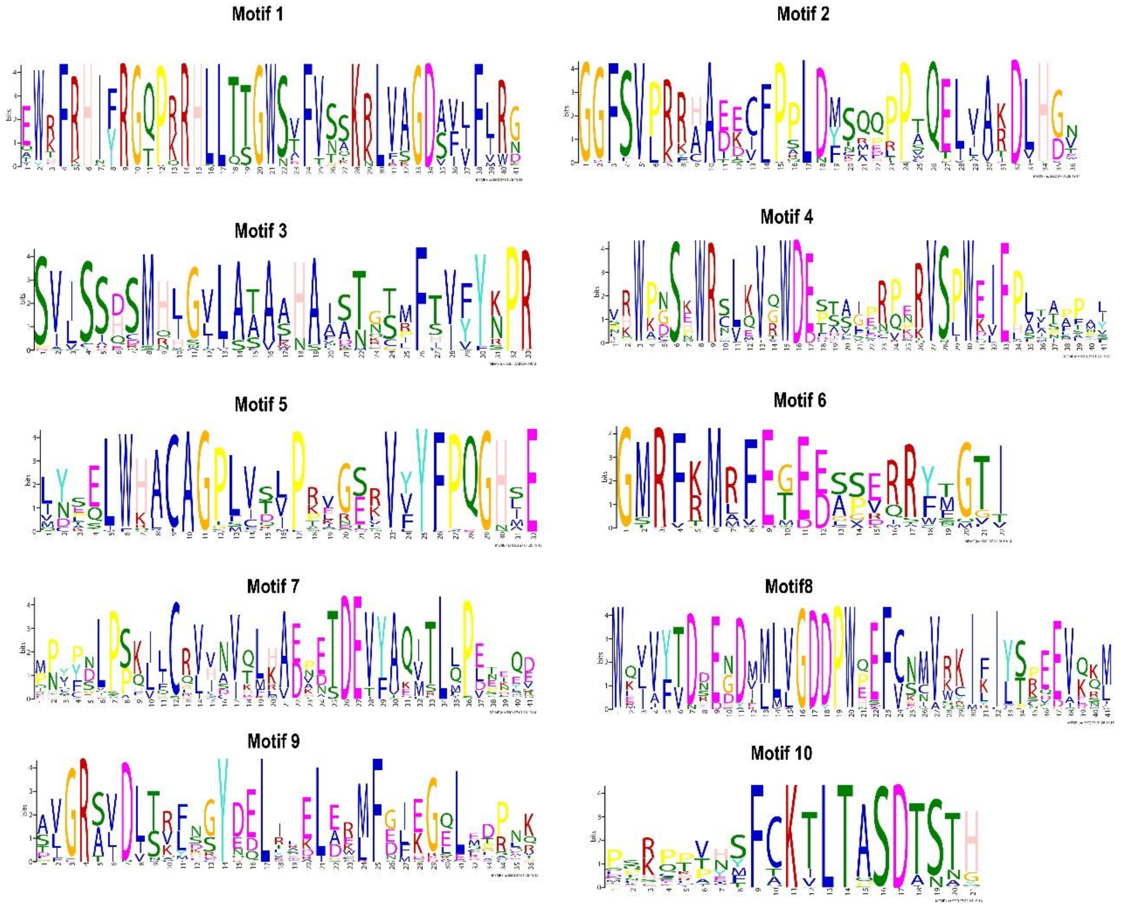

Figure S2. Investigating motif information in amino acid sequences

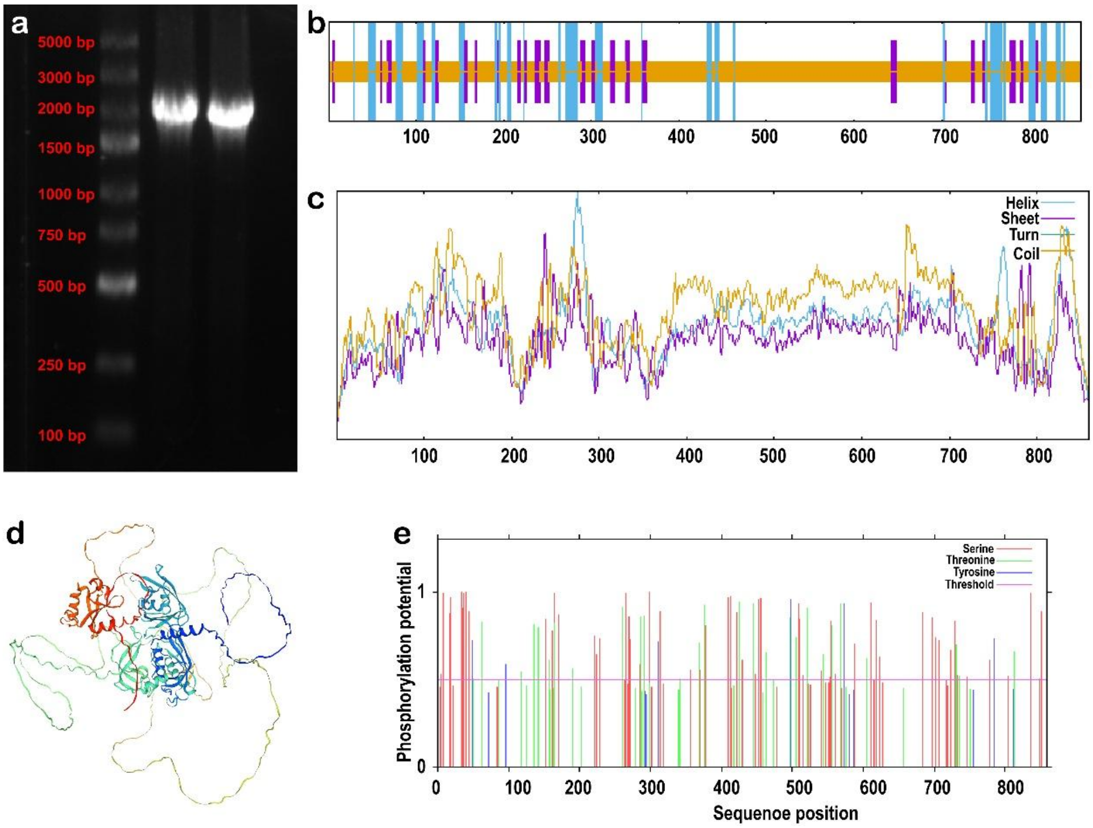

Figure S3. ***McoARF13*** **cloning and bioinformatics analysis. a: PCR product electropherogram, b, c: secondary structure prediction, d: tertiary structure prediction, e: phosphorylation site analysis.**

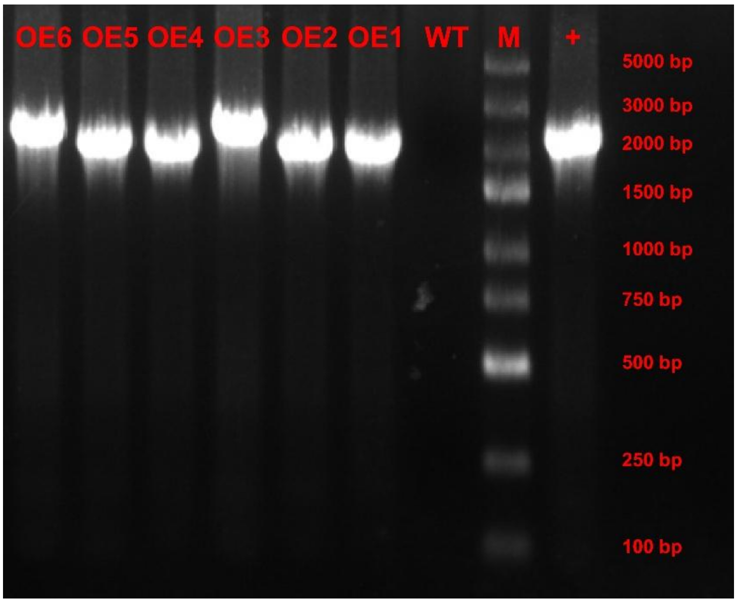

Figure S4. PCR detection of the expression of the positive plant *McoARF13*.
